# HPMC- and PLGA-Based Nanoparticles for the Mucoadhesive Delivery of Sitagliptin: Optimization and In Vivo Evaluation in Rats

**DOI:** 10.3390/ma12244239

**Published:** 2019-12-17

**Authors:** Anroop B. Nair, Nagaraja Sreeharsha, Bandar E. Al-Dhubiab, Jagadeesh G. Hiremath, Pottathil Shinu, Mahesh Attimarad, Katharigatta N. Venugopala, Mohamed Mutahar

**Affiliations:** 1 Department of Pharmaceutical Sciences, College of Clinical Pharmacy, King Faisal University, Al-Ahsa 31982, Saudi Arabia; baldhubiab@kfu.edu.sa (B.E.A.-D.); mattimarad@kfu.edu.sa (M.A.); kvenugopala@kfu.edu.sa (K.N.V.); 2 Department of Pharmaceutics, PA College of Pharmacy, Mangalore 574153, Karnataka, India; drhiremath1975@gmail.com; 3 Department of Biomedical Sciences, College of Clinical Pharmacy, King Faisal University, Al-Ahsa 31982, Saudi Arabia; spottathail@kfu.edu.sa; 4 Department of Biotechnology and Food Technology, Durban University of Technology, Durban 4000, Natal, South Africa; 5 Nova College of Pharmaceutical Education and Research, Jafferguda, Hayat Nagar (Mandal), Telangana 501512, India; principal.pharma.jaff@nova.edu.in

**Keywords:** mucoadhesive, drug release, spray dryer, nanoparticles

## Abstract

Mucoadhesive nanoparticles represent a potential drug delivery strategy to enhance the therapeutic efficacy in oral therapy. This study assessed the prospective of developing HPMC- and PLGA-based nanoparticles using a nanospray drier as a mucoadhesive extended release drug delivery system for sitagliptin and evaluated their potential in an animal model. Nanoparticles were prepared using a Buchi^®^ B-90 nanospray drier. Optimization of particle size was performed using response surface methodology by examining the influence of spray-drying process variables (inlet temperature, feed flow, and polymer concentration) on the particle size. The prepared nanoparticles were characterized for various physicochemical characteristics (yield, drug content, morphology, particle size, thermal, and crystallographic properties) and assessed for drug release, stability, and mucoadhesive efficacy by ex vivo and in vivo studies in rats. A linear model was suggested by the design of the experiments to be the best fit for the generated design and values. The yield was 77 ± 4%, and the drug content was 90.5 ± 3.5%. Prepared nanoparticles showed an average particle size of 448.8 nm, with a narrow particle size distribution, and were wrinkled. Thermal and crystallographic characteristics showed that the drug present in the nanoparticles is in amorphous dispersion. Nanoparticles exhibited a biphasic drug release with an initial rapid release (24.9 ± 2.7% at 30 min) and a prolonged release (98.9 ± 1.8% up to 12 h). The ex vivo mucoadhesive studies confirmed the adherence of nanoparticles in stomach mucosa for a long period. Histopathological assessment showed that the formulation is safe for oral drug delivery. Nanoparticles showed a significantly higher (*p* < 0.05) amount of sitagliptin retention in the GIT (gastrointestinal tract) as compared to control. The data observed in this study indicate that the prepared mucoadhesive nanoparticles can be an effective alternative delivery system for the oral therapy of sitagliptin.

## 1. Introduction

Diabetes mellitus is a metabolic disorder affecting public health worldwide. According to the World Health Organization (WHO) report, 422 million people have diabetes worldwide, particularly in low- and middle-income countries [1]. Typically, this chronic disorder is characterized by elevated levels of blood glucose, which can lead to serious complications in essential systems in the body, including various organs like the heart, as well as blood vessels and nerves. Among diabetes, Type 2 diabetes comprises ~95% of people with diabetes globally, and is largely the result of obesity and insufficient physical activities.

Sitagliptin, a dipeptidyl peptidase-4 (DPP-4) enzyme inhibitor, is indicated for the management of glycemic control in type 2 diabetes mellitus, together with a controlled diet and physical activity. It is one of the most active chemical moieties to treat type-2 diabetics and is a highly selective DPP-4 inhibitor. This incretin enhancer helps to decrease the postprandial and fasting glucose blood sugar level by enhancing the active levels of incretin peptides, such as glucose-dependent insulinotropic peptide and glucagon-like peptide-1 [2]. Studies in humans have signified that the frequent administration of sitagliptin in oral therapy can inhibit plasma DPP-4 activity and significantly increase glucagon-like peptide-1 concentration. The pharmacokinetics of sitagliptin after administering a single dose (100 mg) by oral therapy in healthy humans shows rapid absorption with an absolute bioavailability of ~87% and peak plasma concentrations are reached 1–4 h post-dose [3]. On the other hand, the apparent terminal half-life of sitagliptin in humans is reported to be 8–12 h [4]. In this context, an optimal sitagliptin blood level which can extend the therapy for the whole day could be ideal for this chronic disorder. Therefore, a drug delivery system which can provide gradual release of sitagliptin at the absorption site and can improve its gastrointestinal tolerability and decrease the adverse effects is advantageous. Moreover, an extended release dosage form of sitagliptin could also provide a steady plasma drug concentration and equal effectiveness, which in turn can lead to an improvement in glycemic control and glycemic variability, clinical efficacy, and patient adherence. Indeed, such extended delivery of sitagliptin could be more beneficial in patients with uncontrolled levels of high blood sugar, as continuous availability of sitagliptin may help in the constant production of insulin from the pancreas. Thus, it is postulated that developing a delivery system of sitagliptin that could be substantially retained in the gastrointestinal tract (GIT) and able to steadily release the drug for an extended period of time would be more beneficial than administering it as single dose of 100 mg.

A mucoadhesive drug delivery system provides various advantages by targeting and localizing the nanoparticles at a target site, by decreasing variation in drug level in the plasma, and by extending residence time at the confined target site of drug penetration, and thereby reducing side effects [5,6,7]. Nanoparticles offer promising drug delivery technology by improving the therapeutic window and increasing their tolerability and enhancing their efficacy in the body. Moreover, they help to improve the bioavailability of medications by protecting them from exposure to stomach acid and pepsin. The literature signifies that studies have also been carried out to enhance the therapeutic efficacy of various drugs by formulating mucoadhesive nanoparticles intended for oral therapy [8,9,10]. During the past few decades, various sitagliptin-loaded polymeric nano formulations for oral drug delivery have been developed to enhance bioavailability [2,11,12].

Various methods are available for the preparation of polymeric nanoparticles, such as solvent diffusion, dialysis, microemulsion, interfacial polymerization, supercritical fluid technology, water-in-oil, salting-out, nanoprecipitation, solvent evaporation, and surfactant-free emulsion [13]. However, the main limitations of these approaches are the residual organic solvents, which are utilized in many of the steps, from synthesis to the final product. Complete elimination of the solvents from the product is not possible due to the physical and chemical barriers, even after the product is freeze-dried or dried at elevated temperature. Moreover, the final product may become contaminated by residual solvent from drug-excipient interaction, packaging, or in the accelerated decomposition of the formulation. Manufacturers are responsible for minimizing the organic solvent within the specified limits due to toxicological reasons and due to the lack of therapeutic benefits [14]. The development of a spray-drying technology is not new as far as the drug manufacturing industries are concerned. Recently, spray-dried powders have been receiving increased attention by various research groups. The constant efforts in this field during the last decade have resulted in the conversion of conventional oral tablets to new drug delivery via nanoparticles.

Currently, there are a number of techniques to prepare nanosized particles, i.e., wet-mills, high-gravity controlled precipitation reactors, and homogenizers [15,16]. However, none of these methods produce free-flowing powder, and the final product is still in a suspension form with a wide size distribution. In this context, the patented technology of the Nano Spray Dryer B-90 is a simple and unconventional methodology of producing drug nanoparticles with a narrow particle size range, which is ideally a few nanometers to micrometers, with a high production yield in smaller quantities. Nanoparticles are typically solid in nature and have particles in the range of 10 nm to 1000 nm [17]. The active pharmaceutical ingredients are dispersed, dissolved, adsorbed, or attached to the polymeric matrix system, depending upon the formulation technique [18,19].

Hydroxypropyl methylcellulose (HPMC) is hydrophilic with rapid swelling due to its ability to absorb water, and considered a first generation mucoadhesive polymer (adheres to the mucus non-specifically) that is non-toxic, biocompatible, and biodegradable [20,21]. It acts as a drug-dispersing and viscosity-modifying agent and is a controlled delivery component in oral formulations [22]. Poly (lactic-co-glycolic acid) (PLGA), a pharmaceutical biodegradable polymer approved by the Food and Drug Administration (FDA), can control the drug release in vivo, and can also be used to encapsulate many active pharmaceutical ingredients [23].

In understanding the scientific evidence, experiments were performed by developing and evaluating mucoadhesive sustained release preparations containing blends of two polymers (HPMC, PLGA), and sitagliptin. A review of literature indicates no research explaining the method of formulating HPMC and PLGA nanoparticles by encapsulating sitagliptin. Hence, the aim of this investigation was to develop HPMC- and PLGA-based nanoparticles as a mucoadhesive drug delivery system for sitagliptin and evaluate their potential in an animal model. Optimization of particle size was carried out using response surface methodology by examining the influence of spray-drying process variables (inlet temperature, feed flow, and polymer concentration) on the particle size. Prepared nanoparticles were characterized for physicochemical properties and evaluated for drug release, swelling, mucoadhesion, and stability. In vivo studies were carried out to evaluate the drug distribution in the GIT and compared with oral delivery (suspensions) in Sprague Dawley rats.

## 2. Materials and Methods

### 2.1. Materials

Sitagliptin, HPMC (K100M), and Poly (lactic-co-glycolic acids) (PLGA 50:50; inherent viscosity = 0.40 dL/g) were commercially procured from Sigma Aldrich Co. (St. Louis, MO, USA). All other chemicals used were reagent grade.

### 2.2. Optimization and Formulation of Nanoparticles

The optimization of particle size was carried out using response surface methodology to optimize spray-drying parameters of the inlet temperature (80–120 °C), feed flow (25–50%), and polymer concentration (0.25 to 0.75%), which were coded into −1 to +1 as the limit of the design. The limits of inlet temperature, feed flow, and polymer concentration were selected based on the preliminary studies. The design suggested the experiment run 20 times with the assignment of six center points and six axial points. The drying gas flow rate was maintained at 100 L/min. Sitagliptin (500 mg) was added to the previously dissolved materials consisting of 100 mL of HPMC and PLGA solution. The viscosity of the prepared solution was determined and found to be ~9 cps. A spray nozzle of 5.5 µm was used in the preparation. The filtered solution was used to avoid any nozzle blockage while spraying. The spray-dried particles were scraped out manually from the particle collecting chamber using a rubber scraper [14].

### 2.3. Nanoparticle Yield

The percentage yield was determined by dividing the total weight of nanoparticles obtained after spray-drying by the initial amount of polymer and drug and multiplying by 100, as below: 


Yield = (Weight of nanoparticles obtained)/(Weight of the polymer + drug) × 100.

### 2.4. Drug Content

The method of measuring the percentage of sitagliptin content in the nanoparticle formulation was carried out according to an earlier published method, with a slight alteration [18]. Briefly, the samples were dipped in ethanol–water binary mixture (40:60, 25 mL) and mixed using a vortex mixer for 24 h. Then, the samples were further centrifuged, and the supernatant liquid was filtered using a syringe membrane filter (0.2 µm, Millipore Corporation, New Bedford, MA, USA). The resulting samples were analyzed spectrophotometrically at 430 nm (UV–1601 Spectrophotometer, Shimadzu, Kyoto, Japan) [24]. The analytical method showed good linearity in the range of 1 to 35 μg/mL. The LOQ (limit of quantitation) and LOD (limit of detection) of this method were 0.28 μg/mL and 0.85 μg/mL, respectively.

### 2.5. Scanning Electron Microscopy (SEM)

The surface morphology of the nanoparticles was inspected using SEM (JSM-6390, JEOL, Tokyo, Japan) by placing the powder on the stubs using double-sided adhesive tape, followed by platinum sputtering at 15 kV, using different magnifications between 1000× and 5000×.

### 2.6. Particle Size Distribution

The obtained nanoparticles were added to 0.5 mL n-Hexane and sonicated for 15 min. The mixture of 1.5 mL was poured into the cell and loaded into the Zetasizer Nano ZS (Malvern-Instruments Ltd., Malvern, UK) to assess the particle size distribution. The experiments were carried out in triplicate.

### 2.7. Differential Scanning Calorimetry (DSC) and X-ray Diffraction (XRD)

Differential scanning calorimetry (DSC) generated for pure drug, PLGA, physical mixtures of drug + PLGA + HPMC (1:1:1), and formulations was done by using DSC apparatus (DSC 823, Mettler Toledo, Greifensee, Switzerland) with liquid nitrogen. In short, around 3–5 mg samples were precisely weighed into standard aluminum pans. The empty pan was taken as a reference. The samples were scanned at a rate of 10 °C/min, from 10 °C to 230 °C. X-ray diffraction (XRD) patterns of pure drug, PLGA, physical mixtures of drug + PLGA + HPMC (1:1:1), and formulations were generated using an X-ray diffractometer (Bruker AXS GmbH, Karlsruhe, Germany). The X-ray tube was used at 40 kV and a current of 30 mA. The diffraction angle (2θ) of scans was performed between 0 to 70 °C [25].

### 2.8. Drug Release Pattern and Kinetics

In Vitro sitagliptin release studies were carried out using a customized horizontal glass diffusion tube and cellulose dialysis membrane (MWCO 12–14 kDa, Spectra/por^®^ Spectrum Laboratories Inc., Rancho Dominguez, Berkeley, CA, USA) [26]. The tubes with 100 mg of nanoparticles were attached to the dissolution apparatus and allowed to stir at 100 rpm in 250 mL of gastric fluid (pH 1.2) kept at 37 ± 0.5 °C. At different time intervals (0.2 h, 0.5 h, 1 h, 4 h, 6 h, 8 h, 10 h, and 12 h), a 5 mL sample was removed from the dissolution apparatus and substituted with 5 mL of fresh medium. Samples were filtered using a syringe membrane filter (0.2 μm) and readily analyzed spectrophotometrically at 430. A blank experiment was carried out under similar experimental conditions without the drug. The formulation was further fitted to different kinetic models using SigmaplotInc (Version 10).

### 2.9. Histopathological Examination

The rabbits were anesthetized and the GIT mucosal membrane was excised (IAEC/SSP/19/PR-024). The mucosal tissue samples were pressed between the pads and weighed. Nanoparticles containing 100 mg of the drug were applied on the mucosa and kept for 12 h. The samples were fixed using neutral buffered formalin, then fixed in paraffin and later sectioned into 4 µm thick sections using a microtome. The mucosal membrane was examined under light microscopy (Magnus MX21i, Bio scientific solutions, Chennai, India) after staining the tissue sample with hematoxylin and eosin [27]. The severity of the histopathologic changes was compared with normal GIT mucosal membrane of rabbits.

### 2.10. Drug Retention in GIT

Male Sprague Dawley rats (450–500 g) were subjected to on-demand feeding prior to experimentation for a period of 20–24 h. The ethical guidelines for the animal experiments were followed according to the Institutional Animal Ethics Committee (IAEC/SSP/19/PR-025). Five groups of rats were formed by randomly selecting rats for each group for in vivo experimentation. The rats in four groups were given nanoparticles of sitagliptin orally, while one group was administered an aqueous suspension of the drug. The drug nanoparticles were administered by suspending 50 mg of the drug in 1 mL of saline solution and giving it orally. After the drug administration, six rats were taken after an interval of 0.5 h, 1 h, 3 h, 5 h, 8 h, 10 h, and 12 h and were sacrificed for tissue extraction and analysis. The tissues from the stomach and intestine were extracted and dissected to obtain the inner mucosal surface which bore the nanoparticles of the drug. This mucosal surface was scraped to obtain the residues and the mixture was then homogenized and sitagliptin was extracted. The extracted drug was centrifuged (805× *g* for 20 min) and the supernatant was analyzed spectrophotometrically at 430 nm. The difference in the percentage of drug retention between groups was analyzed by GraphPad Prism (Version 5, Graphpad software, San Diego, CA, USA), and values showing *p* < 0.05 were considered significant.

### 2.11. Stability Studies

The prepared nanoparticles were kept in sealed polyethylene bottles (30 mL) and stored for 12 months. The stability studies of the nanoparticles were performed according to International Council for Harmonization (ICH) guidelines for long-term studies at 30 °C ± 2 °C/65% relative humidity (RH) ± 5% RH. The particle size and drug content of the formulation were examined [28].

## 3. Result and Discussion

The yield of the preparation by spray-drying was 77 ± 4% and other conventional methods reported yields of less than 60% [29]. The drug content was measured to be 90.5 ± 3.5%. The traditional methods reported that the drug content was in the range of approximately 18–47%.

### 3.1. Optimization and Formulation

**Figure 1:**
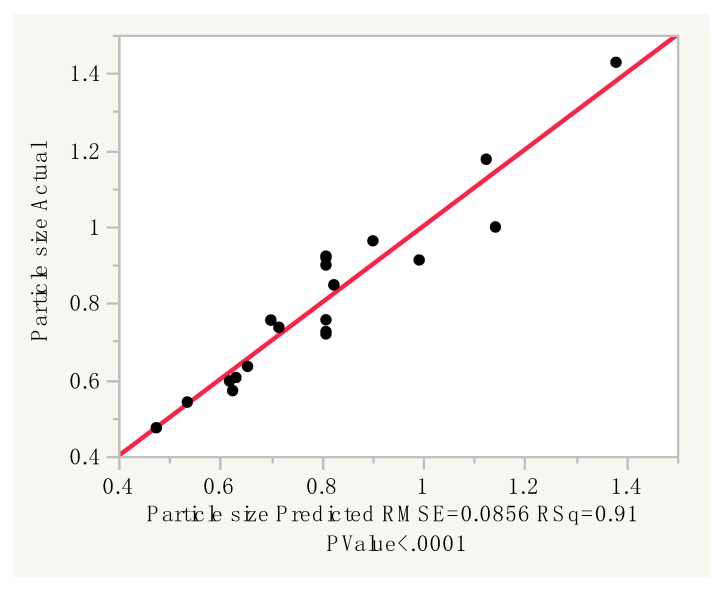
Particle size predicted response and actual results.

The optimization of particle size was modeled on response surface methodology (RSM) using the central composite design (CCD). Particle sizes varied in the range of 0.474–1.428 microns, while the desirable size suggested by the design of experiments software was 0.35–0.60 microns. A linear model was suggested by software to be the best fit for generated design and values, as there was a strong correlation between the model’s predictions and the actual results (R^2^ = 0.91) (Figure 1).

Analysis of Variance (ANOVA) revealed that such a model was reliable for the prediction of particle size (*p* < 0.0001). The model was further confirmed to be adequate as it satisfied the lack of fit test (*p* = 0.57) (Table 1), and the analysis of residual by predicted plot and studentized residual showed no significant errors.

#### 3.1.1. Prediction Expression

The mathematical relationship between three variables (rate, temperature, and polymer concentration) is: 

Particle size = 0.89 + 0.06 * [(Feed flow − 37.5)/12.5] − 0.11 * [(Temperature − 100)/ 20) + 0.20 * (Polymer concentration − 0.5)/0.25] + (Feed flow − 37.5)/12.5 * (Temperature − 100)/20 * −0.03 + (Feed flow − 37.5)/12.5 * (Polymer concentration − 0.5)/ 0.25 * 0.04 + (Temperature − 100)/ 20 * (Polymer concentration − 0.5)/ 0.25 * −0.14.

#### 3.1.2. Pareto Plot of Estimates

**Figure 2:**
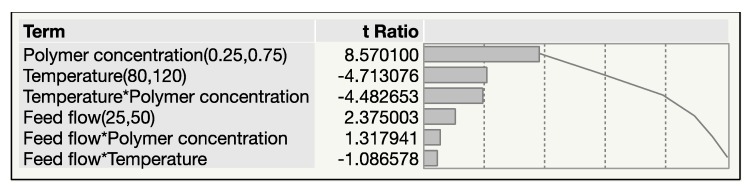
Pareto chart response bar indicating the factors has a direct or counter effect on response. * Mathematical expressions, denoting multiplication.

According to the Pareto chart, the weight of polymer concentration, temperature, and feed flow ranges from largest to smallest, respectively. From the chart, statistically significant parameters can be selected for analysis of the surface plot and contour plot to determine the response (Figure 2).

Based on the prediction expression while one independent variable was kept at a fixed value, the surface plot and contour plots illustrating the interplaying roles of other two variables affecting the response were constructed, leading to a deeper understanding of the conditions at which the experiment should be held for optimized particle size to be generated.

**Figure 3:**
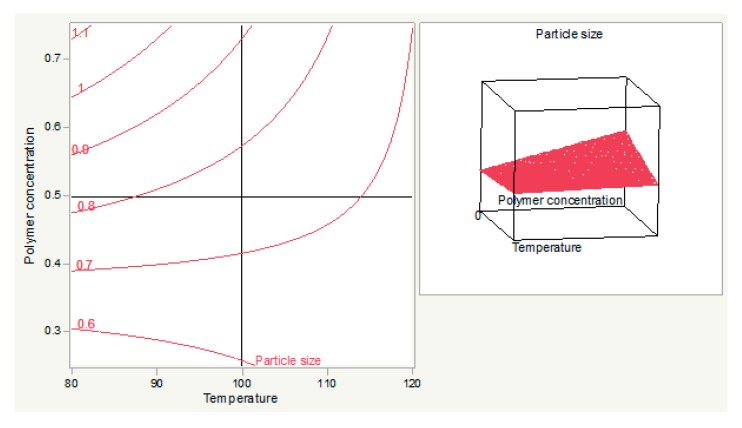
The relationship between polymer concentration and temperature (**left**) and its response surface graph (**right**).

The relationship between the polymer concentration and the temperature is shown when feed flow was set to 10. As concentration has the most significant effect, as indicated by the Pareto chart, particle size increases greatly in response to a little increase in this factor. The expected size (<0.38 microns) was obtained at the temperature <104.63 °C and polymer concentration <0.6% (Figure 3).

**Figure 4:**
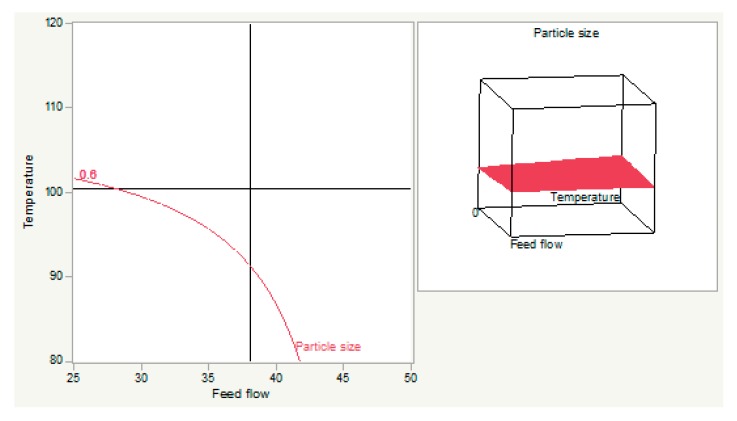
The relationship between temperature and feed flow (**left**) and its response surface graph (**right**).

The relationship between the feed flow and temperature is shown as polymer concentration, which was set at 0.5 microns. An increase in temperature and feed flow resulted in an increase in particle size. With temperature <101.58 °C and feed flow <41.84%, the expected particle size could be achieved (Figure 4).

**Figure 5:**
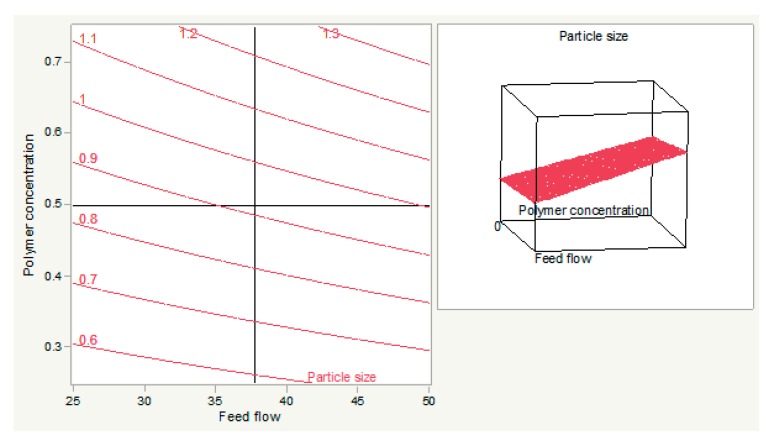
The relationship between polymer concentration and feed flow (**left**) and its response surface graph (**right**).

The relationship between the polymer concentration and feed flow is shown when the temperature was set at 150 °C. The same effect of concentration is shown here as the previous plot. An increase in polymer concentration and feed flow increased the size of the particle. The desired size cannot be achieved at feed flow >42.10% and concentration >0.31 microns (Figure 5).

### 3.2. Surface Morphology

**Figure 6:**
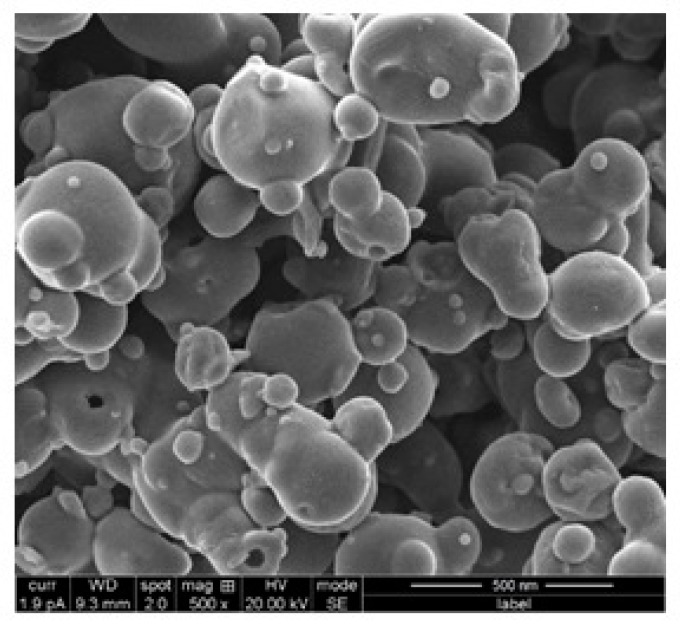
A representative scanning electron microscopy micrograph of spray-dried nanoparticles.

Spray-dried formulations require a one-step process to produce dried particles in comparison to conventional techniques. Spray-drying resulted in an irregular (shriveled) shape of the particles, which is moderately different from our earlier studies, where the polymer used was albumin or chitosan [11,12]. This caused shriveled shapes because sprayed drops came in contact with the hot air in the drying chamber, which resulted in the particle swelling, shrinking, or twisting. Observation of Figure 6 indicates that the particles were agglomerated, but that could be overcome by evaporating more moisture content from the liquid feed by decreasing the flow rate. Moreover, due to the outlet temperature, the heat transfer was high during spray-drying for the collected particles. The lower the outlet temperature, the higher the water content of the dried particles, which would improve the particles’ morphologies [30]. The surface properties of the pure drug and polymers were compared with reported literature and our formulations were found to be shriveled [31,32].

### 3.3. Particle Size Distribution

**Figure 7:**
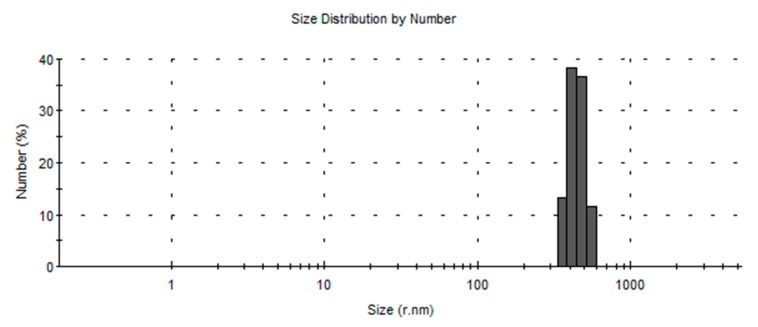
A representative size distribution image of prepared nanoparticles.

The prepared nanoparticles showed a narrow particle size distribution with an average particle size of 448.8 nm (Figure 7). The particle size observed here is also comparable with the earlier studies [11,12]. Narrow size distribution will help to maintain constant plasma levels within the therapeutic window for a longer time and will decrease incidents of toxicity or underexposure, which is vital for any dosage form. The narrower sized particle was due to the piezoelectric actuator being driven in the range of 60 kHz, causing the perforated stainless-steel membrane to vibrate and eject lots of equal sized droplets every second, leading to a submicron size distribution [33].

### 3.4. DSC

**Figure 8:**
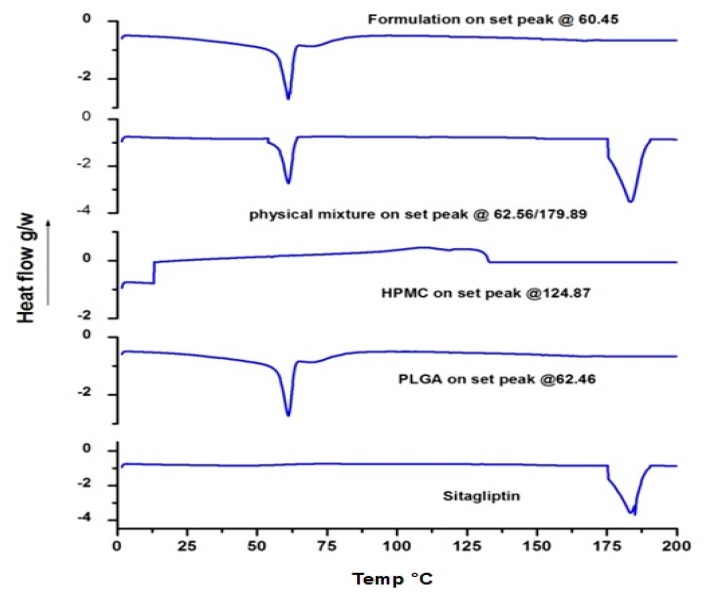
A representative differential scanning calorimetry (DSC) thermogram of sitagliptin, poly (lactic-co-glycolic acid) (PLGA), physical mixture and formulation.

Figure 8 shows the DSC thermograms of sitagliptin, PLGA, physical mixture, and formulation. The thermogram of the sitagliptin endothermic peak generated 180.78 °C. The endothermic peak of pure PLGA, HPMC, and physical mixture was observed at 62.46 °C, 124.87 °C, and 62.56/179.89 °C, respectively. The formulation showed only a polymeric peak at 60.45 °C. This might be due to the effect of dissolution of a certain amount of added sitagliptin in the polymer. The drug peak, however, did not appear in the formulation, which may be due to sitagliptin being entrapped in an amorphous state or a dissolved state.

### 3.5. XRD

**Figure 9:**
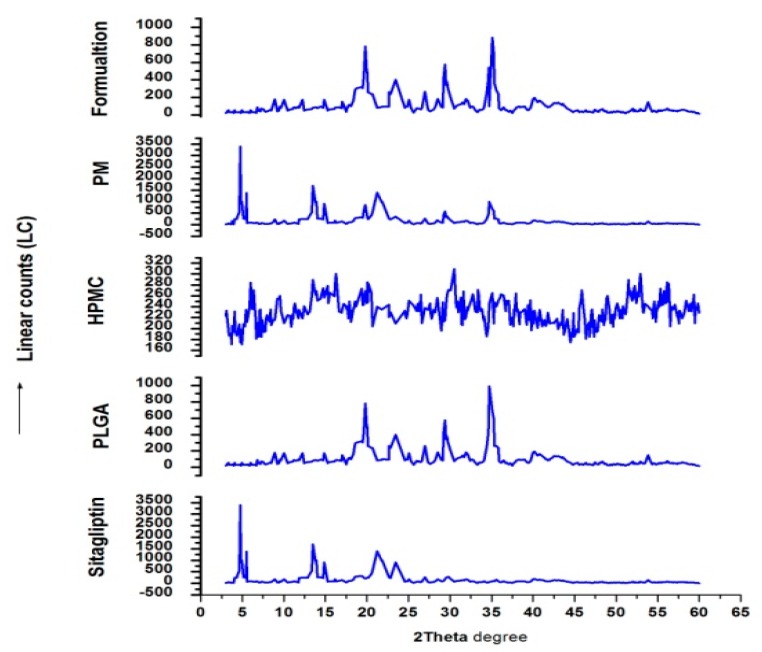
A representative X-ray diffraction patterns of sitagliptin, PLGA, hydroxypropyl methylcellulose (HPMC), physical mixture of drug and polymers, and formulation.

The nature or internal physical stage of sitagliptin in the nanoparticles was also assessed by XRD, which is a great tool to assess the crystalline lattice arrangement. Figure 9 shows the crystallographic characteristics of sitagliptin, PLGA, HPMC, physical mixture of drug and polymers, and formulation. The X-ray diffractograms of pure drug intensity peaks were identified, sitagliptin showed intensive peaks at 2θ scattered angles of 4.91°, 16.37°, 18.27°, 19.05°, 20.29°, 22.27°, 23.12°, 24.76°, 25.98°, 29.93°, 30.03°, while PLGA showed peaks at 8.89°, 10.02°, 12.24°, 19.79°, 23.24°, 25.02°, 29.41°, HPMC at 30.56°, and 52.93°; mixtures, however, gave peaks at 3.78°, 5.50°, 8.72°, 13.49°, 13.90°, 14.8°, 21.20°, 21.81°, 29.63°, and 34.73° 2θ degrees. Drug-loaded formulation intensive peaks were identified at 8.86°, 12.24°, 19.78°, 24.12°, 25.02°, and 27.01° 2θ degrees. The drug peaks disappeared in formulations. This infers that the drug may be present in a dissolved/amorphous state.

### 3.6. Drug Release and Kinetics

**Figure 10:**
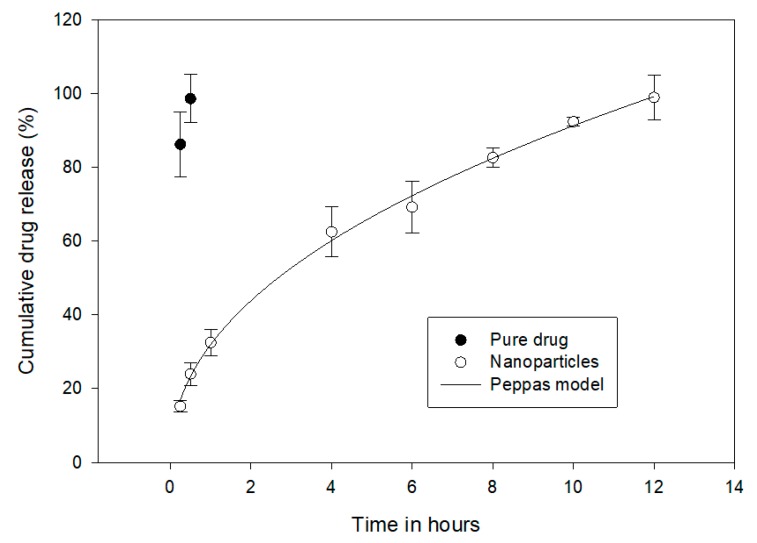
Comparison of percentage sitagliptin released from nanoparticles and pure drug (control).

Figure 10 shows the drug release for the pure drug and nanoparticle formulation. The pure drug was rapidly released within the first 30 min (98.6 ± 6.5%). The drug release was extended in the nanoparticle formulation. It was 98.9 ± 6% at 12 h, indicating that the polymer greatly influenced the rate of drug release, which was a biphasic release with an initial burst release of 24.0 ± 3.1% at 30 min, due to the presence of uncovered drug particles on the external surface of the nanoparticles, and the remaining continued to release in a sustained manner (98.9 ± 6%) up to 12 h in simulated intestinal fluid. This proves the ability of the polymer to retain intact in both acidic and basic simulated fluid. Results of in vitro release studies were then fit with various kinetic models [34]. The most studied models are the Higuchi, first-order, Hixon–Crowell, Baker–Lonsdale, and Peppas, and the best fit with R^2^ = 0.9972 was seen in the Peppas model, with release exponent value n = 0.72. This kinetic model suggested that the release of sitagliptin from prepared nanoparticles is diffusion controlled. In addition, the observed release exponent n indicates that the diffusion mechanism from nanoparticles is anomalous transport.

### 3.7. Swelling

**Figure 11:**
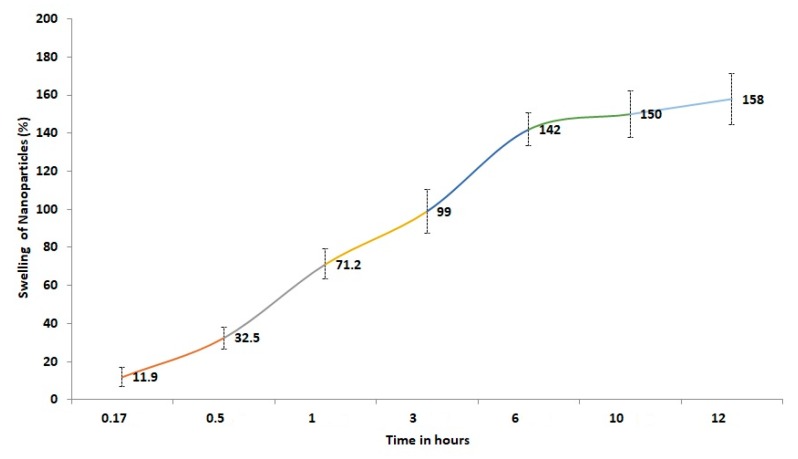
The percentage swelling pattern of prepared nanoparticles for a period of 12 h.

Swelling of the formulation facilitates and supports its adhesion along mucous layers. The swelling profile of nanoparticles was ~150 times their initial size (Figure 11). The swelling mechanism was due to polymer ionization, which led to nanoparticle swelling. Also, this swelling was due to water uptake by the nanoparticles and by saturation of microcapillary voids within the nanoparticles, as reported earlier [35].

### 3.8. Mucoadhesion

Mucoadhesion of the formulation in the stomach area will provide retention of drug and its subsequent release. Mucoadhesive properties were measured for the nanoparticles in the stomach and were found to be 52% ± 3% in 4 h, indicating that they could reside in the stomach for a prolonged period. This is probably due to the HPMC polymer, being both mucoadhesive and a viscosity modifier [21,22]. Literature indicates that the nanoparticles prepared using mucoadhesive polymers are usually adhered in the surface of mucosal membrane due to an adhesion mechanism [36].

### 3.9. Drug Retention in GIT

**Figure 12:**
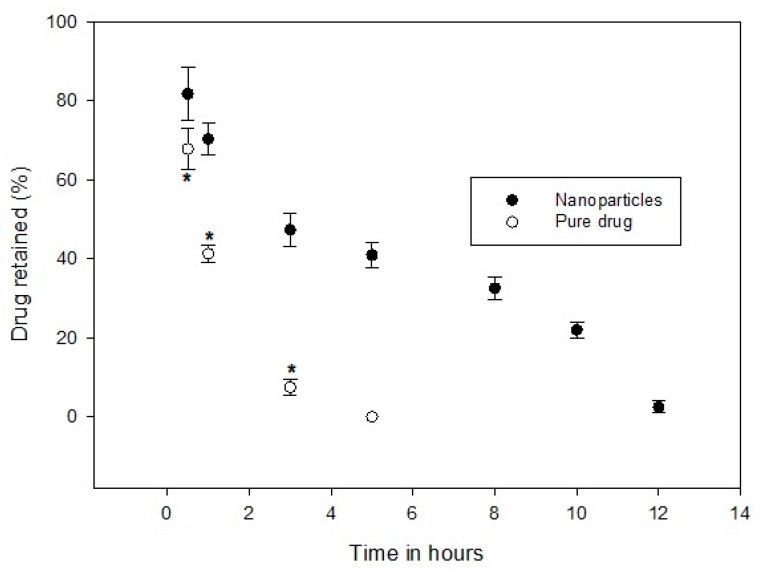
Comparison of the amount of sitagliptin retained in the rat gastrointestinal tract after the administration of nanoparticles and pure drug (control). The data represents average ± SD (*n* = 6). The values of the control group are statistically different (*) when compared to the nanoparticle treated group at *p* < 0.05.

In Vivo studies were performed to assess the mucoadhesion property of prepared nanoparticles in Sprague Dawley rats [37]. The amount of sitagliptin adhered in the GIT membrane at various time intervals was determined after oral administration of prepared nanoparticles and compared with control (suspension of pure sitagliptin) (Figure 12). It is apparent from Figure 12 that nanoparticles showed a significantly higher (*p* < 0.05) amount of sitagliptin retention in the GIT as compared to the concentration of aqueous suspension of sitagliptin (Control). In the case of nanoparticles, the percentage of drug retained at time intervals of 0.5 h, 1 h, 3 h, 5 h, 8 h, 10 h, and 12 h was found to be 81.75%, 70.32%, 47.33%, 40.95%, 32.56%, 21.98%, and 2.49%, respectively. Indeed, such a retention profile is ideal for the prolonged release of sitagliptin by extending the residence time of nanoparticles in the GIT. Moreover, the values observed here also signify that the combination of HPMC and PLGA nanoparticles can provide greater drug retention than the chitosan nanoparticles [12]. Alternatively, the percentage of drug retained in the GIT drastically decreased and was significantly low (*p* < 0.05) when administered as a suspension. Noticeably, the suspension showed a short residence time, as no detectable amount of sitagliptin was seen at 5 h. These results indicate that the fusion or adsorption of sitagliptin was greater in the nanoparticles. The possible explanation for this observation is that the mucoadhesive polymer (HPMC) used in preparing nanoparticles helps in adhering them due to their inherent chemical nature. Being a hydrophilic polymer, HPMC provides rapid swelling of the nanoparticles, which also contributes to higher mucoadhesion.

### 3.10. Histopathology

**Figure 13:**
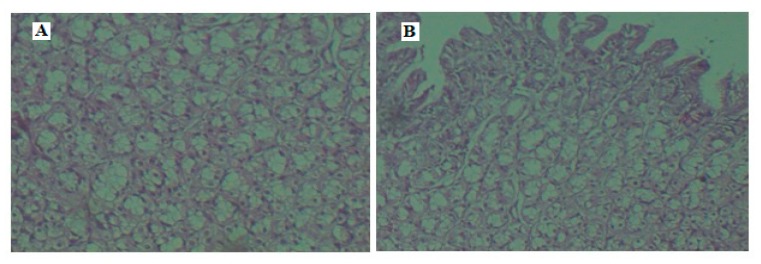
Photomicrographs of rabbit GIT mucosal membrane after oral drug administration of (**A**) nanoparticles and (**B**) normal control.

Histopathological examination was carried out to assess the changes in the rabbit GIT mucosal membrane following oral therapy of nanoparticles and compared with normal tissue. Photomicrographs in Figure 13 signify that the GIT mucus membrane of the nanoparticle-treated group had no difference when compared with the normal rabbits. Moreover, no inflammation or variation in histological architecture was noticed in the nanoparticle-treated groups. Hence, the mucoadhesion of prepared nanoparticles does not affect the mucus membrane and can be considered safe for oral administration.

### 3.11. Stability

**Figure 14:**
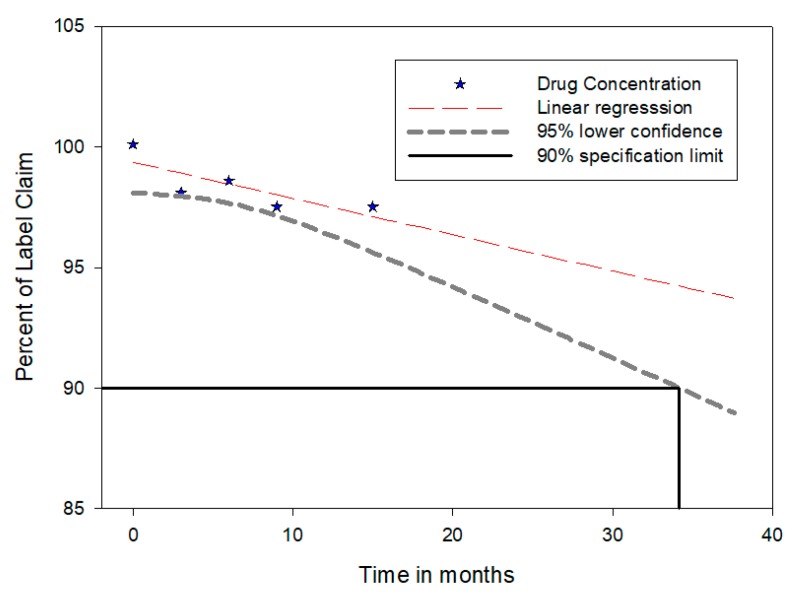
Shelf-life data of sitagliptin nanoparticles.

After storing the nanoparticle powder according to ICH guidelines for long-term studies at 30 °C ± 2 °C/65% RH ± 5% RH, the nanoparticles were aggregated. The size and the drug content were found to be 598.2 nm and 89.5% ± 2.5%, respectively. The shelf-life estimation was made using sigma plot 10 software to analyze the stability of the product (Figure 14). It is apparent from the Figure 14 that the drug concentrations observed (shown as solid star) at various time intervals had a linear regression line. The 95% lower confidence line is also presented in Figure 14. In general, the shelf life of the product was determined as the x axis coordinate for the intersection of the 95% lower confidence line with 90% drug activity. Therefore, the intersection determined exactly by the macro in this case was 30.8 months, as evidenced in Figure 14.

## 4. Conclusions

Optimized nanoparticles were successfully prepared using mucoadhesive (HPMC) and controlled release (PLGA) polymers. The data observed here indicate that spray-drying process variable influenced the particle size of nanoparticles. The physicochemical properties of nanoparticles were found to be ideal for oral mucoadhesive delivery. Ex vivo and in vivo data substantiated the objective of preparing mucoadhesive nanoparticles, as demonstrated by the prolonged release of sitagliptin by extending the residence time of nanoparticles in the GIT, which, in turn, can improve its therapeutic efficacy in oral therapy. In a nutshell, HPMC- and PLGA-based nanoparticles appear to be an important and promising formulation approach to enhance the clinical efficacy of therapeutic actives.

## Figures and Tables

**Table 1:** Summary of results of variance analysis.

Source	Degree of Freedom	Sum of Squares	Mean Square	F Ratio
**Model**	6	0.9103593	0.151727	20.7187
**Error**	13	0.0952013	0.007323	Prob > F
**Total**	19	1.0055606	0.159050	<0.0001 *
**Lack of Fit**	8	0.04569042	0.005711	0.5768

* Significant difference in particle size prediction.
